# Whole-Genome Multi-omic Study of Survival in Patients with Glioblastoma Multiforme

**DOI:** 10.1534/g3.118.200391

**Published:** 2018-09-18

**Authors:** Yeni L. Bernal Rubio, Agustin González-Reymúndez, Kuan-Han H. Wu, Corinne E. Griguer, Juan P. Steibel, Gustavo de los Campos, Andrea Doseff, Kathleen Gallo, Ana I. Vazquez

**Affiliations:** * Department of Epidemiology and Biostatistics; † Institute for Quantitative Health Science and Engineering; ** Department of Animal Science and Department of Fisheries and Wildlife; †† Department of Statistics and Probability; ‡‡ Department of Physiology; §§ Department of Pharmacology and Toxicology, Michigan State University, East Lansing, Michigan, 48823; ‡ Department of Public Health Sciences, Henry Ford Health System, Detroit, Michigan, 48202; § Department of Neurosurgery, University of Alabama at Birmingham, Birmingham, Alabama, 35294

**Keywords:** Gene expression, glioblastoma multiforme, methylation, prognosis, single nucleotide polymorphism

## Abstract

Glioblastoma multiforme (GBM) has been recognized as the most lethal type of malignant brain tumor. Despite efforts of the medical and research community, patients’ survival remains extremely low. Multi-omic profiles (including DNA sequence, methylation and gene expression) provide rich information about the tumor. These profiles are likely to reveal processes that may be predictive of patient survival. However, the integration of multi-omic profiles, which are high dimensional and heterogeneous in nature, poses great challenges. The goal of this work was to develop models for prediction of survival of GBM patients that can integrate clinical information and multi-omic profiles, using multi-layered Bayesian regressions. We apply the methodology to data from GBM patients from The Cancer Genome Atlas (TCGA, n = 501) to evaluate whether integrating multi-omic profiles (SNP-genotypes, methylation, copy number variants and gene expression) with clinical information (demographics as well as treatments) leads to an improved ability to predict patient survival. The proposed Bayesian models were used to estimate the proportion of variance explained by clinical covariates and omics and to evaluate prediction accuracy in cross validation (using the area under the Receiver Operating Characteristic curve, AUC). Among clinical and demographic covariates, age (AUC = 0.664) and the use of temozolomide (AUC = 0.606) were the most predictive of survival. Among omics, methylation (AUC = 0.623) and gene expression (AUC = 0.593) were more predictive than either SNP (AUC = 0.539) or CNV (AUC = 0.547). While there was a clear association between age and methylation, the integration of age, the use of temozolomide, and either gene expression or methylation led to a substantial increase in AUC in cross-validaton (AUC = 0.718). Finally, among the genes whose methylation was higher in aging brains, we observed a higher enrichment of these genes being also differentially methylated in cancer.

Glioblastoma multiforme (GBM) is a locally intracranial malignant tumor, which can develop *de novo* or through progression from low-grade astrocytomas (Zhen *et al.* 2010). GBM has been recognized as the most common and deadly type of malignant brain tumor in adults (Louis *et al.* 2007), characterized by tumors growing and spreading rapidly into brain tissue (Barnett 2007). Moreover, GBM is characterized by a low prognosis, with an overall median survival time of 15 months for patients (Martinez *et al.* 2010). Major challenges of GBM therapies are related to the absence of an effective treatment due to their complex and heterogeneous biology, as well as to the extensive genetic and biological variability in how aggressively the tumor behaves (Furnari *et al.* 2007). This scenario highlights the importance of assessing the genomic factors that affect time to death, given the lack of understanding of genetic aspects related to the progression of the disease (Bondy *et al.* 2008).

The increasing availability of high-dimensional omic data, such as DNA data (*e.g.*, Single Nucleotide Polymorphism, SNP), epigenetic data (*e.g.*, DNA methylation) and transcriptomic data (*e.g.*, mRNA quantification) makes it possible to explain the inter-individual variation in patients outcome (*e.g.*, time to survival) and to identify molecular characteristics potentially associated with how aggressive the tumor is. Thus, different studies have implemented models for integration of molecular predictors, aiming to determine the genomic factors affecting the development of tumors and the prediction accuracy of long-term survival in GBM patients (Zhao *et al.* 2015; Lu *et al.* 2016). However, there is not sufficient information to assess the proportion of variance in disease risk and prognosis on GBM that can be explained by molecular predictors and their interaction with demographical covariates such as age, race, and sex. In addition, joint modeling of omics and clinical covariates can lead to redundancy of information, an issue that has not been well explored. We previously proposed a model integrating whole-genome multi-omic profiles for gaining insights of the biology and potentially also for prediction of disease risk (Vazquez *et al.* 2014). We successfully used this model to assess inter-individual variation in breast cancer progression (Vazquez *et al.* 2016). Recently, the model was extended to incorporate gene expression-by-treatment interaction (González-Reymúndez *et al.* 2017). Therefore, novel statistical toolkits available provide unique opportunities to determine the variation in patient outcomes that can be explained by different layers of omics and potentially predict outcomes in GBM patients.

One important unaccounted aspect in multi-omic integration models for GBM is the treatment which affects the overall survival of the patients and remains based on surgery, radiation and concomitant daily chemotherapy using temozolomide (Chen *et al.* 2013). Several studies have evaluated the methylation status of the gene encoding the repair enzyme O6-methylguanine-DNA methyltransferase, *MGMT*, considered a biomarker of GBM patient response to temozolomide. However, all patients with GBM continue to be treated with temozolomide, regardless of *MGMT* methylation status (Hegi *et al.* 2005). This context suggests that information from chemotherapy treatments can be integrated with omic profiles in order to evaluate the therapeutic benefits from different treatment protocols.

The goal of this study was to evaluate demographical and clinical covariates, treatments and omics from the tumor, and their association with variability in survival on GBM patients. We implemented different multi-omic integration models, assessing the relationship between clinical covariates, treatments (temozolomide and radiation), and high-dimension molecular omics with survival time, for identification of clinical and molecular factors affecting survival rate and prediction accuracy of cancer outcomes, in a cohort of glioblastoma multiforme patients from The Cancer Genome Atlas (TCGA).

## Materials and Methods

Data consisted of 502 GBM patients, after edition, was made available by The Cancer Genome Atlas (TCGA). Data included information from clinical and demographic variables, treatments, and high-dimensional omics data collected from normal and tumor tissue. Only the following group of patients were included in the study: “*de novo*” GBM, either African-American or European-American origin, complete vital status and known treatment. The sample described belongs to the post-edited set. Different subsets of the data were analyzed with different omics arrays; thus analyses vary in sample size. Details of the omics data utilized, quality control and corrections are detailed in the Supplementary File S1.

### Baseline model

We first assessed the effects of demographics and clinical covariates on survival using a Cox regression. The covariates tested included: sex (306 males and 196 females), race (23 African American and 470 European American), age at diagnosis (modeled as a factor with four levels, (<50 years, n = 131; 51-60 years, n = 138; 61-69 years, n = 125 and > 69 years, n = 108), diagnosis method (tumor resection, n = 437; biopsy, n = 65), use of radiation (yes, n= 103; no, n = 399) and of chemotherapy, and tumor purity. For chemotherapy we consider both a dummy variable (yes, n = 353; no, n = 149) as well as covariates representing the use of specific drugs (*e.g.*, temozolomide) and combinations of drugs. Tumor purity was quantified using the consensus measurement of purity estimations (CPE) derived by Aran *et al.* (2015).

We regressed survival time on the covariates described above using the coxph function of the Survival regression R-package (Therneau and Grambsh 2000). Since only age at diagnosis and use of temozolomide (yes: n = 298; no: n = 204) had significant effects on survival time, our baseline model used those two covariates.

### Assessment of the association Between omics and clinical and demographic covariates

Omic profiles are not independent of demographic covariates (Spiegl-Kreinecker *et al.* 2015). Understanding these associations is essential before incorporating demographics to omics in a time-to-death predictive model. Therefore, before combining omics with clinical covariates we assessed the association between omics and covariates by regressing demographics and other covariates on the first 10 principal components (PCs) derived from each of the omics. We report results from these regression analyses as well as graphical summaries of the PCs and their association with covariates, since different tumors may have omic-specific characteristics depending on the subject’s race, age or gender.

### Gene set enrichment analysis

Gene set enrichment analysis was performed for genes with differential methylation between younger and older patients, and previously reported sets, including: (1) a set of genes reported to methylate while brains age (Horvath *et al.* 2012), as well as (2) a set of genes reported as hyper-metylated in GBM tumors (Lai *et al.* 2014). Differential methylation of a gene was evaluated with a linear model for methylation M-values from each probe, as a function of the age of the patient. The raw *p*-values from *t*-tests were adjusted using False Discovery Rate (Benjamini and Hochberg 1995). Genes with q-value of less than 0.05 were declared significant. Next, we did a gene set enrichment analysis to determine whether the genes more strongly associated with age were indeed over-represented by previously reported genes differentially methylated with brain aging, (Horvath *et al.* 2012) and more importantly, with genes hyper-methylated in GBM tumors (Lai *et al.* 2014). The brain aging set included 111 genes and the GBM hyper-methylated set included 273 genes (Supplementary File S2), and there were no overlapping genes between these two sets. The enrichment analysis evaluated the *t*-statistics distribution of all the genes tests with the distribution within the specific set of genes reported for brain aging and for GBM methylation. An over-representation of high or low *t*-values in the specific sets indicates enrichment. The enrichment analyses were implemented using the limma R package (Ritchie *et al.* 2015).

### Integration of omic profiles for cancer prognosis

Subsequently, we extended the baseline model by adding omic information. We did this by adding either one omic at a time or in some cases combinations of omics. All the omics considered are high-dimensional; therefore, we introduced them as random effects in a (log-normal) Bayesian survival model similar to the one used by Vazquez *et al.* (2016) and González-Reymúndez *et al.* (2017) for multi-omic prediction of breast cancer outcomes. In our Bayesian model the logarithm of survival time was modeled as a function of the covariates of the baseline model plus omics. Considering two omics sets, the regression took the form:

$$y_{i}=\mu +\underset{j}{\sum }x_{ij}\alpha_{j}+\underset{k}{\sum }z_{ik}\beta_{k}+\underset{l}{\sum }w_{il}\gamma_{l}+\varepsilon_{i}$$

where $y_{i}$ is the logarithm of survival time, μ is an intercept, $\underset{j}{\sum }x_{ij}\alpha_{j}$ represents a linear regression on the covariates of the baseline model, $\underset{k}{\sum }z_{ik}\beta_{k}$ and $\underset{l}{\sum }w_{il}\gamma_{l}\;$ represent regression terms for two different omics, here $z_{ik}$ and $w_{il}$ are features in each of the omic sets (*e.g.*, $z_{ik}$ may represent CNV at the *k^th^* CNV site and $w_{il}$ may be the expression of the *l^th^* gene in the *i^th^* subject) and $\varepsilon_{i}$ is an error term assumed to be *IID* (identically and independently distributed) normal with null mean and variance $\sigma_{\varepsilon }^{2}$.

#### Bayesian Likelihood:

For subjects with observed survival time, the conditional distribution of the data given the parameters and covariates was normal with mean $\eta_{i}=\mu +\underset{j}{\sum }x_{ij}\alpha_{j}+\underset{k}{\sum }z_{ik}\beta_{k}+\underset{l}{\sum }w_{il}\gamma_{l}$ and variance $\sigma_{\varepsilon }^{2}$, that is $p\left(y_{i}|\eta_{i},\sigma_{\varepsilon }^{2}\;\right)=N\left(y_{i}|\eta_{i},\sigma_{\varepsilon }^{2}\right)$. For right censored data, survival time is unknown and the likelihood function was $p\left(y_{i}|\eta_{i},\sigma_{\varepsilon }^{2}\;\right)=\text{Φ}\left(\frac{y_{i}-\eta_{i}}{\sigma_{\varepsilon }\;}\right)$, where $y_{i}$ was follow-up time and Φ(.) is the cumulative distribution function of a standard normal random variable. Therefore, the joint Bayesian likelihood was

$$p\left(y|\alpha ,\beta ,\gamma ,\sigma_{\varepsilon }^{2}\right)=\overset{n}{\underset{i=1}{\prod }}\underset{observed}{\underset{︸}{N{\left(y_{i}|\eta_{i},\sigma_{\varepsilon }^{2}\right)}^{1-c_{i}}}}\underset{censored}{\underset{︸}{\text{Φ}{\left(\frac{y_{i}-\eta_{i}}{\sigma_{\varepsilon }\;}\right)}^{c_{i}}}}.$$

Above, $c_{i}$ is an indicator variable taking a value of 1 for right censored observations and 0 otherwise.

#### Prior distribution:

The covariates of the baseline model were treated as “Fixed” effects; therefore, the $\alpha_{j}’s$ were assigned flat priors; this yields estimates that are equivalent to maximum likelihood. Omics are high-dimensional; therefore, we assigned to these effects priors that induce shrinkage of estimates. There is a vast array of priors that can be used (de los Campos *et al.* 2013). Among these, the Gaussian prior induces shrinkage of estimates and it is particularly well suited for complex traits that are affected by large number of omic factors. Furthermore, the Gaussian prior offers important computational advantages because it enables using the “Kernel trick” (Shawe-Taylor and Cristianini 2004), that is, representing the model with a number of unknowns that is proportional to the number observations. This is particularly useful in a setting like ours where the number of features (*e.g.*, methylation sites) is considerably larger than the sample size. Based on these considerations and following Vazquez *et al.* (2016), we assigned IID normal priors to omic effects. Specifically, we assumed $\alpha_{j}\begin{array}{c} IID\\ ∼ \end{array}N\left(0,\sigma_{\alpha }^{2}\right)$ independent of $\gamma_{j}\begin{array}{c} IID\\ ∼ \end{array}N\left(0,\sigma_{\gamma }^{2}b\right)$. Since the Gaussian distribution is closed under linear operations we have that the vectors $u_{z}=\left\{u_{zi}=\underset{k}{\sum }z_{ik}\beta_{k}\right\}$ and $u_{w}=\left\{u_{wi}=\underset{l}{\sum }w_{il}\gamma_{l}\right\}$ follow multivariate normal distributions of the form $u_{x}∼N\left(0,K_{x}\sigma_{\alpha }^{2}\right)$ and $u_{w}∼N\left(0,K_{w}\sigma_{\gamma }^{2}\right)$. Here, $K_{x}=XX^{'}$ and $K_{x}=WW^{'}$ are kernel matrices that describe similarity between subjects based on their omic profiles. Therefore, we represented the model in terms of $u_{x}$ and $u_{w}$.

The Bayesian model is completed by assigning priors to variance parameters. For these we choose independent scaled-inverse Chi-square prior, that is $p\left(\sigma_{\varepsilon }^{2},\sigma_{\alpha }^{2},\sigma_{\gamma }^{2}\right)=\chi^{-2}\left(\sigma_{\varepsilon }^{2}|S_{\varepsilon },df_{\varepsilon }\right)\chi^{-2}\left(\sigma_{\alpha }^{2}|S_{\alpha },df_{\alpha }\right)\chi^{-2}\left(\sigma_{\gamma }^{2}|S_{\gamma },df_{\gamma }\right)$. Here, $S_{.}$ and $df_{.}\;$ are scale and degree of freedom hyper-parameters, respectively.

Combining the likelihood and the prior, and using Bayes theorem, the posterior distribution becomes

$$p\left(\mu ,u_{x},u_{w}\sigma_{\varepsilon }^{2},\sigma_{\alpha }^{2},\sigma_{\gamma }^{2}|y\right)\propto \overset{n}{\underset{i=1}{\prod }}\underset{observed}{\underset{︸}{N{\left(y_{i}|\eta_{i},\sigma_{\varepsilon }^{2}\right)}^{1-c_{i}}}}\underset{censored}{\underset{︸}{{\left(1-\text{Φ}\left(t_{i}|\eta_{i},\sigma_{\varepsilon }^{2}\right)\right)}^{c_{i}}}}\;\times \;N\left(u_{x}|0,K_{x}\sigma_{\alpha }^{2}\right)\times \;N\left(u_{w}|0,K_{w}\sigma_{\gamma }^{2}\right)\chi^{-2}\left(\sigma_{\varepsilon }^{2}|S_{\varepsilon },df_{\varepsilon }\right)\chi^{-2}\left(\sigma_{\alpha }^{2}|S_{\alpha },df_{\alpha }\right)\chi^{-2}\left(\sigma_{\gamma }^{2}|S_{\gamma },df_{\gamma }\right)$$

This posterior distribution does not have a closed form. However, samples from the posterior distribution can be obtained using a Gibbs sampler. We used the BGLR software (Pérez and de los Campos 2014) to draw samples from the posterior distribution of the above model.

Convergence was assessed by inspecting the trace plot of variance parameters. We used the coda R-package to evaluate the Monte Carlo error, making sure that for all variance parameters the MC error was smaller than 1% of the estimated posterior mean of each parameter. In our analyses, we used the default settings of BGLR that consists of assigning variance parameters weakly informative chi-square priors, see Pérez and de los Campos (2014) for further details.

#### Sequence of models:

We fitted the Bayesian model above described with those clinical covariates that had significant effect on survival, with covariates plus one omic, and with covariates plus several omics. From these analyses, we report the proportion of variance explained by each set of predictors along with measures of goodness of fit and model complexity.

#### Prediction accuracy:

We assessed prediction accuracy using 5-fold cross-validations with subjects randomly assigned to folds. The cross validation was repeated 20 times for each of the models. From the CV we computed the Receiver Operating Characteristic (ROC) curve and the area under that curve (AUC), *e.g.*, Robin *et al.* (2011). ROC curves and AUC were computed using the pROC R-package package (Robin *et al.* 2011).

### Data Availability

The results presented in this manuscript are in whole based upon data generated by the TCGA Research Network through the National Cancer Institute Genomic Data Commons portal: https://portal.gdc.cancer.gov/. Omic datasets (DNA Methylation, CNV, gene expression) as well as clinical and demographic covariates are available for download. Also, open access data can be accessed through the web-based GDC Data Portal (IRB permission number 15-827). Source code is available at the Supplementary File S3 and at the website https://github.com/anainesvs/glioblastoma. Supplemental material available at Figshare: https://doi.org/10.25387/g3.7092146 and https://doi.org/10.25387/g3.7096346.

## Results

A total of 502 patients identified as *de novo* GBM and diagnosed between 2008 and 2015 were included in this study. European American patients comprised 93.6%, while African American patients corresponded to 6.4%. 61% of patients were male, while 39% were female. Time to death was observed in 75.1% of the patients, while 24.9% (n = 125) were considered right censored at the last follow-up time recorded. Also, 52.3% of the patients had survival of less than one year. The overall median (± SD) follow-up time was 9.3 months (±14.8 months) for all patients, 7.9 months (±11.8 months) for censored patients, while the median survival time for uncensored patients was 10.4 months (±15.7 months). The mean (± SD) age at diagnosis was 58.2 years (±14.06 years) for all patients. 26.1% of patients had less than 50 years, 27.5% between 50 and 60 years, 24.9% between 60 and 69 years and the remaining 21.5% were older than 69 years. Finally, 70.3% (n = 353) of patients received chemotherapy and 29.2% (n = 103) received chemotherapy and also radiation. Within the group of patients receiving chemotherapy, 84.4% (n = 298) received temozolomide. In this study, 18% of patients older than 61 years received radiotherapy, while 30% of the patients younger than 61 years received radiation.

### Characteristics of omics profiles by demographics

**Figure 1:**
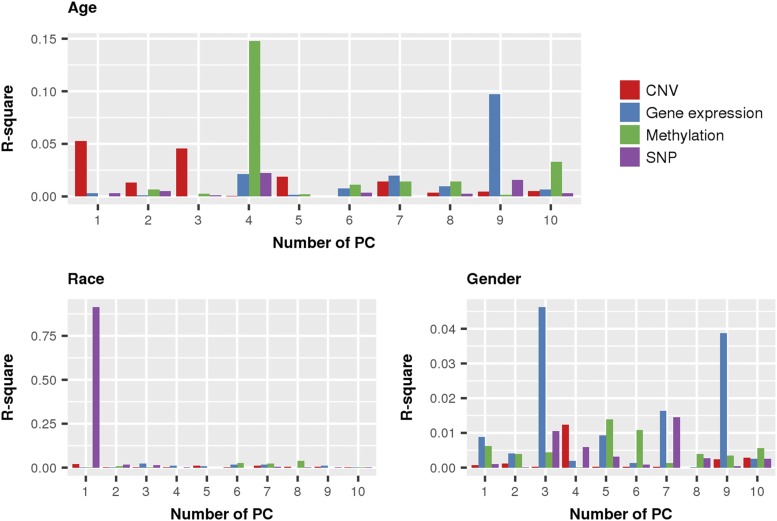
Regression of principal components derived from similarity matrices on demographical covariates. The first ten principal components (PC, x-axis) were obtained from regression of SNP, methylation, gene expression and CNV on the clinical covariates of: A) age, B) race, and C) sex. The proportion of variance explained is presented in terms of R-square (y-axis).

**Figure 2:**
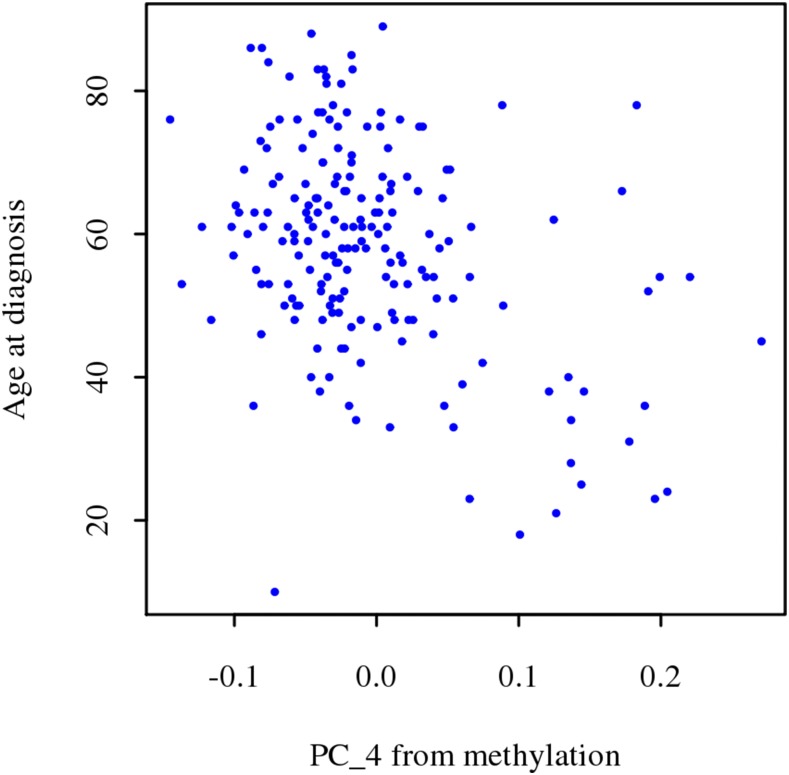
Fourth (x-axis) principal component derived from methylation *vs.* age at diagnosis (y-axis).

Age, race, and sex of the patients may produce effects in tumors. The association between omic-derived principal components (PC) and demographic covariates is shown in Supplementary Figures S1 to S12, revealing omic-based differences in the tumors. Figure 1 shows the proportion of variation in omic-derived PC explained by covariates. Age was significantly associated with the fourth and tenth PC derived from methylation (Figure 2), explaining 18% (14.8% and 3.3% for PC four and ten, respectively) of the variation in methylation (Supplementary Table S1). Higher values at the fourth eigenvector are associated with increments in age at diagnosis. Additionally, the first and third CNV-derived PC were also significantly associated with age at diagnosis, which explained 9.9% of the variation in CNV in GBM patients (5.3% and 4.6% explained by PC1 and PC3, respectively; Supplementary Table S2).

**Figure 3:**
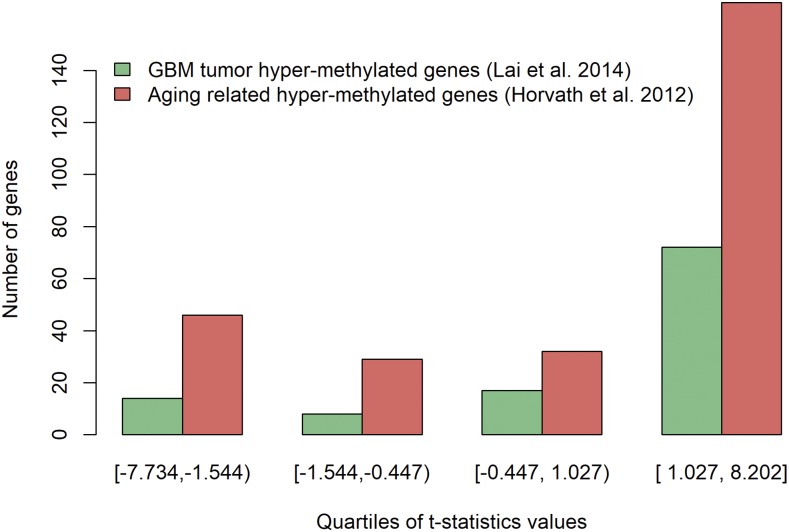
Overlap between previously reported hypermethylated genes (involved in GBM and brain aging) and genes differentially methylated with age in our dataset. The *t*- values from tests of differential methylation with age where split in four quartiles. The green and red bars represent the number of genes in each quartile overlapping with previously reported hypermethylated genes: involved in GBM (Lai *et al.* 2014 – green bars), and in brain aging (Horvath *et al.* 2012– red bars).

The enrichment analysis suggested that our set of genes that are significantly methylated with age overlaps with genes previously known as involved in aging and /or GBM tumor process (Figure 3). The list is over-represented for pathways involved in the correct development and differentiation of the nervous system (activation of *HOX* clusters, neural crest, ectoderm formation; Supplementary Figure S13). Moreover, the list is also enriched for targets of transcription factors that play a role during neural crest development and differentiation, such as *FOXB2*, *BARX1* and *GSC*. The top ten most significant genes with this pattern were hypermethylated with age in GBM patients. The list contains *RUNX3* (a transcription factor that functions as a tumor repressor (Chi *et al.* 2017), *HCN1* (that codes for a proton channel that regulates the normal brain rhythmic activity (Pan *et al.* 2015), and the fork-head transcription factors *FOXD3* (involved in cell pluripotency and reported as tumor suppressor; Li *et al.* 2017), and *FOXA1* (reported as tumor progressor in several human cancers; Lin *et al.* 2018).

 Other demographic covariates associated with differences in the omics included race and gender. The first SNP-derived PC was significantly associated with race, which explained 91.3% of the variation in the SNP set (Supplementary Table S3). It has been widely reported that race can be identified with the first principal components derived from common SNPs (*e.g.*, Campbell *et al.* 2005), even if the SNPs are not specifically ancestry informative markers. Additionally, sex was the most significant covariate associated with gene expression, explaining 8.5% of the variation in gene expression levels between GBM patients (4.6% and 3.9% for PC three and nine respectively; Supplementary Table S4). This association remained even after sexual chromosomes were discarded from the SNP set evaluated. A significant association between gene expression and sex was also reported by expression of different regulators. Survival in males and females is determined by the expression of different regulators and signaling pathway components (Yang *et al.* 2017). Nonetheless, gender or race were not significant for survival time.

### Age and temozolomide are significant prognostic predictors of survival

Survival analyses aiming to identify relevant clinical and treatment covariates were performed. We found that age of diagnosis and the use of temozolomide had a statistically significant effect on survival. Notably, our data showed that survival of GBM patients decreased by 2.82% per year of age (Table 1). Patients treated with temozolomide had a risk of death 1.56 times lower than patients treated with a different drug (*P* < 0.0001).

**Table 1:** Survival analysis based on clinical and demographical covariates. Hazard ratio, confidence interval (95%) and p-values for clinical and treatment covariates for GBM patients (n = 502)

Covariate ^1^	Hazard ratio (95% CI)^2^	Pr(>|z|)^3^
**Age at diagnosis**	1.029 (1.020,1.038)	< 0.0001
**Gender (male)**	1.064 (0.859, 1.319)	0.5684
**Race (European-American)**	1.128 (0.709, 1.795)	0.6119
**Method of diagnosis (tumor resection)**	1.194 (0.877, 1.625)	0.2591
**Temozolomide**	0.641 (0.496, 0.828)	0.0007
**Bevacizumab**	0.317 (0.078, 1.281)	0.1069
**Radiation**	0.918 (0.612, 1.377)	0.6793
**Temozolomide + bevacizumab**	2.731 (0.644, 11.576)	0.1727
**Temozolomide + radiation**	0.839 (0.483, 1.459)	0.5360
**Bevacizumab + radiation**	0.463 (0.181, 1.187)	0.1089

1 Clinical covariates included in the model; ^2^Hazard ratio and corresponding 95% confidence interval; ^3^*p*-value for significance of the covariate.

Survival curves of the significant covariates showed the highest mortality in groups that were not treated with temozolomide or among the older patients (Supplementary Figure S14). The effect of anti-cancer drugs such as lomustine and temozolomide resulted as significantly associated to survival in GBM patients (Supplementary Table S5). However, fewer patients were treated with lomustine (n = 45) in comparison to temozolomide (n = 298). Tumor purity, defined as the estimated proportion of cancer cells in the sample (Mean and SD 0.8565 and 0.096 respectively), was also non-significant for patient survival time. Therefore, models generated in the following sections are extensions from a model including age and temozolomide, and were considered for estimation and assessment of prediction accuracy. Finally, neither the use of bevacizumab or radiation, nor the pairwise interactions between these two drugs with temozolomide were significantly associated with overall survival of GBM patients (Table 1).

### Methylation profiles capture relevant proportion of inter-individual variation in survival

Inter-individual variation in survival time explained by different factors was evaluated in patients with complete omic information (n = 191). Models including age and temozolomide have a residual variance of approximately 0.22 smaller than the model with only a common intercept (Table 2). Among omics, the larger inter-individual variation in survival was explained by methylation (0.876) followed by gene expression (0.726), SNP (0.657) and CNV (0.264). We found that CNV alone explains 14.9% of the inter-individual variation in GBM survival after accounting for age and use of temozolomide as chemotherapy treatment. Although the model including only gene expression explained a larger proportion of variance than SNP, the former model also had the largest residual variance (0.724).

**Table 2:** Inter-individual variation in survival, residual variance and Deviance Information Criteria (DIC) for models including age, temozolomide and whole omic profiles. Standard error (se) in parenthesis

Model	Variance explained (se)				Residual variance (se)	DIC
	SNP	Methylation	Gene expression	CNV		
**Intercept**	–	–	–	–	1.14 (0.128)	413.1
**Age at diagnosis**	–	–	–	–	0.998 (0.113)	396.6
**Temozolomide**	–	–	–	–	0.993 (0.111)	398.9
**SNP**	0.657 (0.196)	–	–	–	0.538 (0.188)	386.8
**Methylation**	–	0.876 (0.248)	–	–	0.468 (0.158)	372.9
**Gene expression**	–	–	0.726 (0.281)	–	0.724 (0.155)	401.0
**CNV**	–	–	–	0.264 (0.074)	1.114 (0.094)	873.6
**Age + Temozolomide**	–	–	–	–	0.904 (0.101)	389.7
**Age + Temozolomide + SNP**	0.414 (0.157)	–	–	–	0.521 (0.159)	370.8
**Age + Temozolomide + Methylation**	–	0.527 (0.191)	–	–	0.498 (0.142)	366.3
**Age + Temozolomide + Gene expression**		–	0.371 (0.168)	–	0.684 (0.118)	383.6
**Age + Temozolomide + CNV**				0.149 (0.05)	0.921 (0.077)	824.9
**Age + Temozolomide + SNP +** **Methylation + Gene expression + CNV**	0.166 (0.087)	0.184 (0.101)	0.141 (0.076)	0.108 (0.051)	0.424 (0.117)	331.2

The models that incorporated individual omic sets were also extended for inclusion of age and temozolomide, allowing the reduction of the residual variance as well as the improvement of goodness of fit. Although the models including high-dimensional omic sets may tend to over fit, reducing the residual variance explained by the model, the model including methylation captured the largest proportion of variation among individuals in comparison to the models including gene expression, SNP or CNV. Moreover, after combining predictors in a single model, the highest proportion of inter-individual variation in survival was still explained by the methylation (0.184). This model was characterized by a lower residual variance and better fit (lower DIC) in comparison to models including one predictor.

### Whole-genome omic profiles are predictive of survival time

**Figure 4:**
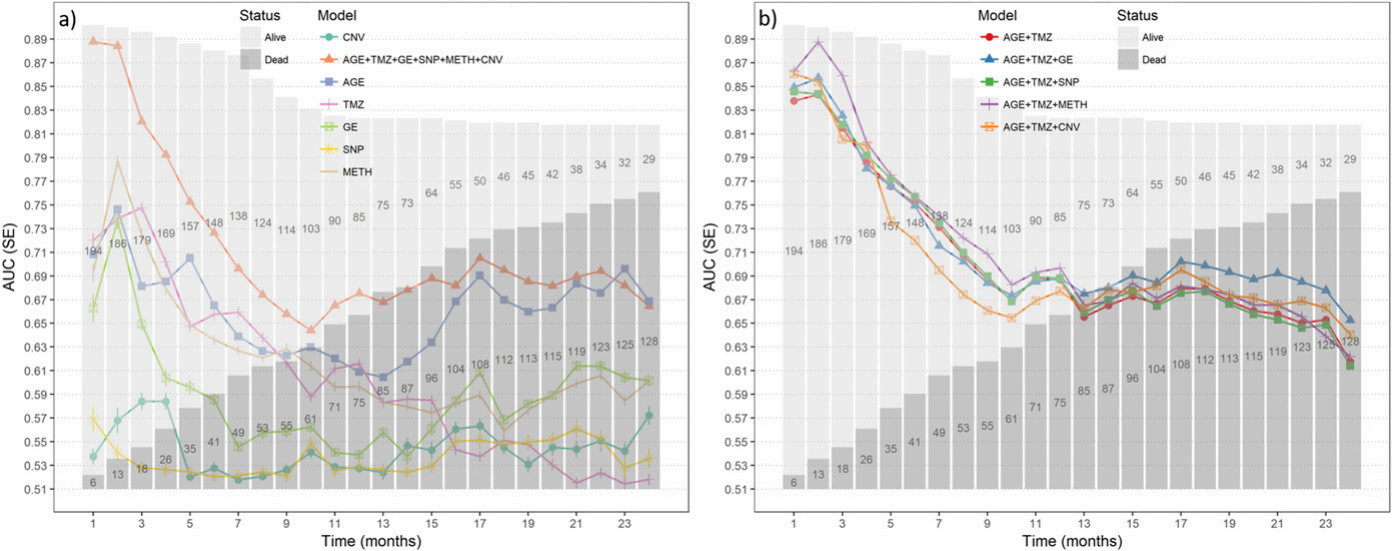
Prediction accuracy of survival time for models including age, temozolomide (TMZ), SNP, methylation (METH), gene expression (GE) and CNV. Prediction accuracy was measured in terms of AUC (y-axis) *vs.* months (x-axis). A histogram of survival time for GBM patients is represented in both figures. Models include: a) single predictors; b) predictors incorporating omic profiles into the baseline model (age + temozolomide).

Predictive ability was evaluated in terms of Area Under the Curve (AUC) by implementing a cross-validation for all single factors and for the integrative model. AUC was estimated with the pair (*y*, $\hat{y}$), where *y* = {0,1} is a variable indicating alive status each month after diagnosis, and $\hat{y}$ is the prediction of the model when the fold containing *y* is removed. The prediction was evaluated first across models with one predictor at a time (Table 3 and Figure 4a), and then in the series of models including clinical covariates and omics (Table 4 and Figure 4b). The model including age, temozolomide, and all omic sets as well as the model that included age, temozolomide and gene expression, and age, temozolomide, and methylation, had similar predictive ability performance, which was also the highest (∼0.72). Lower prediction accuracy was obtained with CNV (0.546) and SNP (0.539).

**Table 3:** Prediction accuracy of single predictors models in terms of AUC from 5-fold 20 CV

Model^1^	Mean AUC^2^	SD^3^	Proportion of times model in row (column) had AUC > model in column (row) over 20 CV											
			Age	Tmz	SNP	Meth	Ge	CNV	Age + Tmz	Age + Tmz + SNP	Age + Tmz + Meth	Age + Tmz + Ge	Age + Tmz + CNV	Age + Tmz + Ge + Meth + SNP + CNV
**Age**	0.664	0.036		0.75	>0.95	0.88	>0.95	>0.95	0.29	0.29	0.21	0.08	0.17	0.08
**Tmz**	0.606	0.075	0.27		0.67	0.42	0.63	0.71	<0.05	<0.05	<0.05	<0.05	<0.05	<0.05
**SNP**	0.539	0.014	<0.05	0.33		<0.05	<0.05	0.50	<0.05	<0.05	<0.05	<0.05	<0.05	<0.05
**Meth**	0.623	0.059	0.13	0.58	>0.95		0.67	>0.95	<0.05	<0.05	<0.05	<0.05	<0.05	<0.05
**Ge**	0.593	0.045	<0.05	0.38	>0.95	0.33		>0.95	<0.05	<0.05	<0.05	<0.05	<0.05	<0.05
**CNV**	0.547	0.020	<0.05	0.29	0.50	<0.05	0.04		<0.05	<0.05	<0.05	<0.05	<0.05	<0.05

1 Models used for analysis, described on Table 2; ^2^Mean of prediction accuracy measured by AUC by using 20 5-fold cross-validations for each model; ^3^Standard deviation of AUC.

**Table 4:** Prediction accuracy of integrative models in terms of AUC from 5-fold 20 CV

Model^1^	Mean AUC^2^	SD^3^	Proportion of times model in row (column) had AUC > model in column (row) over 20 CV											
			Age	Tmz	SNP	Meth	Ge	CNV	Age + Tmz	Age + Tmz + SNP	Age + Tmz + Meth	Age + Tmz + Ge	Age + Tmz + CNV	Age + Tmz + Ge + Meth + SNP + CNV
**Age + Tmz**	0.705	0.063	0.71	>0.95	>0.95	>0.95	>0.95	>0.95		0.42	0.08	0.25	0.38	0.33
**Age + Tmz + SNP**	0.706	0.065	0.71	>0.95	>0.95	>0.95	>0.95	>0.95	0.58		0.08	0.29	0.38	0.33
**Age + Tmz + Meth**	0.717	0.073	0.79	>0.95	>0.95	>0.95	>0.95	>0.95	0.92	0.92		0.50	0.71	0.46
**Age + Tmz + Ge**	0.718	0.058	0.92	>0.95	>0.95	>0.95	>0.95	>0.95	0.75	0.71	0.50		0.71	0.71
**Age + Tmz + CNV**	0.706	0.064	0.79	>0.95	>0.95	>0.95	>0.95	>0.95	0.63	0.63	0.29	0.29		0.29
**Age + Tmz + Ge + Meth + SNP + CNV**	0.715	0.067	0.82	>0.95	>0.95	>0.95	>0.95	>0.95	0.43	0.64	0.37	0.43	0.71	

1 Models used for analysis, described on Table 2; ^2^Mean of prediction accuracy measured by AUC by using 20 5-fold cross-validations for each model; ^3^Standard deviation of AUC.

The single factor with highest prediction accuracy was age at diagnosis followed by methylation, in particular for times greater than four months. Age and temozolomide were similar in prediction accuracy for periods less than ten months. In general, the integrative model outperformed the results obtained from the single-predictor. Prediction accuracy was high for patients with shorter survival time, indicating the challenge in prediction of time to death in patients. Longer survival maybe achieved a number of factors, less aggressive tumors, better response to treatment or earlier detected cancers (n = 256). A better prediction accuracy of short survival time is likely due to omics providing information of elements that separate more aggressive or therapy resistant tumors; however, it could also be an effect of a loss of sample size with time. Figures 4a and 4b show a histogram of the data available each month after diagnosis, overlapped with the prediction accuracy of different models.

Predictive ability obtained with age and methylation showed a similar pattern and it seems clear that aging has association with methylation. We also evaluated the predictive performance of methylation after controlling for age. We found that the mean of prediction accuracy of methylation was 0.69, which was superior than a model including only age (AUC of 0.66; Supplementary Figure S15).

## Discussion

The present work evaluated the contribution of demographical covariates, treatment, and several omics in survival time of GBM patients, and estimated the accuracy of prediction with the different sources of information at all survival times (*i.e.*, patients with short, or long survivals). The prognostic clinical covariates included were age at diagnosis, race, and gender and for modeling treatment indicator variables admitted chemotherapy and radiation.

Among the demographic and treatment factors, the use of temozolomide along with age constituted the most significant (*P* < 0.05) prognosis factors for GBM. Age at diagnosis has been also previous previous reported as a significant covariate for determining survival in GBM patients (Adamson *et al.* 2009; Lu *et al.* 2016), mostly indicating a significant association of long-term survival (>3 years) with young age at diagnosis in GBM patients. Age of the patient has also been suggested as a factor affecting surgical and therapeutic decisions; younger patients can have better tolerance than older patients to aggressive chemotherapy (Wedding *et al.* 2007), leading to a higher survival probability in younger patients. However, treatment and tolerance to chemotherapy are indicated as consistent for all patients (Bozdag *et al.* 2013) and therefore differences in survival according to age should be due to potential differences in molecular profiles of the tumor. In agreement with previous studies (Lu *et al.* 2016), race and gender were not significantly associated with survival in this study. Although association between race and survival time results controversial, it may be possible that recorded differences in survival between races could be explained by differences in socio-economic status and access to health care among African American and European American patients rather than biological differences in the tumors.

We also indagated TCGA data, about the proportion of inter-individual variation in survival explained by different sets of omics, and the prediction accuracy that can be achived with a cross validation. To the best of our knowledge, this study is the first effort for estimating the inter-individual variation that can be explained by molecular-based predictors of clinical outcomes from GBM patients. Whole genome methylation in the tumor was the most informative omic, explaining observed inter-individual variation in survival and predicting data from out-of training patients. Results for methylation holded either when methylation was considered alone, or conditional in age and chemotherapy use. The inter-individual variation explained by me-thylation and predictive ability was higher than the one achieved by whole genome gene expression. Strikingly, gene expression was not in overall a good predictor of survival. Previous studies in breast cancer (Vazquez *et al.* 2016; González-Reymúndez *et al.* 2017) showed a relevant proportion of variation associated to methylation but 33% lower than gene expression, which has shown patterns that can be clustered in tumor subtypes (Perou *et al.* 2000; *e.g.*, absence or presence of hormone receptors). Contrastingly, GBM does not seem to present clear clusters by gene expression. Our results suggest that a better direction to form survival relevant clusters would be exploring methylation-enabled clusters. Finally, SNP and CNV where the sets with lower variance of survival associated as well as prediction accuracy. In previous studies, multiple germline CNVs have been recognized as one of the most prevalent factors predisposing individuals toward cancer (Parker *et al.* 2015). Nevertheless, once the cancer is established, our results indicate that other factors, such as tumor genome-wide methylation, were more informative about prognosis than tumor-specific CNV. Kim *et al.* (2012) also reported poor predictions of long survival in GBM, when using CNV, and also reported lower predictions than those obtained using gene expression. Our study agree with Kim and collaborators, although we densely evaluates survival at all points.

Genetic heterogeneity as well as other omic heterogeneity, may occur between tumors from different groups (*e.g.*, male/female heterogeneity). We inquiry the omics data for potential race, gender and age specific patterns. SNP showed association to race and gene expression with gender and methylation with age. However, while race and gender were not significantly affecting survival, age was a significant factor, and our results indicate that methylation profiles present variation according to age. Previous reports have shown a significant and positive correlation (> 98% of the CpG sites) between DNA methylation levels and age of the human brain (Lai *et al.* 2014). However, our results show that methylation explains survival beyond what is explained by age. After controlling for age, the prediction accuracy of methylation was superior than the accuracy obtained by only age (mean AUC of 0.69 *vs.* 0.66, respectively). Thus, we further studied whether genes that were differentially methylated by age (Horvath *et al.* 2012) were also differentially methylated in tumor *vs.* normal tissue (Lai *et al.* 2014). We have identified that our set of genes significantly methylated with age overlaps with genes previously known as involved in aging and /or GBM tumor process. DNA methylation changes subtly with age; these changes can introduce a high degree of epigenetic variability in aging cells. Such epigenetic phenomena could impact immune response through masking or unmasking potential tissue antigens as well as by regulating the differentiation and response of immune cells (Issa 2003). Aging may also affect the rate at which naive B and T cells are produced as well as the composition and quality of the mature lymphocyte pool (Montecino-Rodriguez *et al.* 2013). The consequences of immune system aging include an increase in autoimmune phenomena, incidence of cancer, chronic inflammation, and predisposition to infections (Ramos-Casals *et al.* 2003). These may be suggesting specific connections by which aging may also be associated to more aggressive tumors. Age at diagnosis was also associated with tumor-specific CNV. The existence of a relationship between age and CNVs in healthy blood cells *in vivo* is in agreement with the association between age and CNV reported in the present study. According to Ramos-Casals *et al*. (2003), this association can also explain the age-related reduction in the diversity of the immune system and the subsequent increments in the predisposition and incidence of infections and complex diseases such as cancer. However, our results use tumor specific CNV.

In sum, the accuracy attained under the model integrating age, temozolomide and methylation as well as age, temozolomide and gene expression and age, temozolomide, gene expression and methylation (model not shown) were superior to simpler models; the superiority was shown as mean prediction accuracy and robustness of the prediction (*i.e.*, number of times the full model(s) were superior than the simpler models without omics or some of the covariates, Table 4). The addition of SNPs and CNVs slightly hurt prediction accuracy. The similar prediction accuracy observed in the models considering methylation and gene expression can be a result of co-variation between omics sets. For instance, methylation and gene expression sets show a moderate correlation (0.36) across the whole profile (results not shown).

Many studies focus on prediction of long term survival after diagnosis of GBM (*e.g.*, >24 months), since characteristics of the less deadly tumors need to be identified to learn the biology of tumor aggression. Our study densely evaluates survival at all points and shows that long-term survival is one of most difficult outcomes to predict. We know that prediction accuracy is better immediately after diagnosis (months 1 to ∼6). The reason for this counterintuitive observation could be due to the nature of the data–where at the beginning of a follow up, sample size is naturally larger–or, it could be that highly aggressive tumors are omic-wise different, and the omic sets capture these differences.

In summary, age and temozolomide treatment are not only significantly associated to survival, but also good predictors. At different ages, however, there are differences in methylation, partially following a well-established relation between age and methylation. Whole genome methylation was identified as the most informative omic of survival followed by gene expression. Our findings admit that in GBM, the inter-individual variation survival were poorly predicted by SNPs and somatic CNVs.
